# Neo-Splicetopes in Tumor Therapy: A Lost Case?

**DOI:** 10.3389/fimmu.2022.849863

**Published:** 2022-02-21

**Authors:** Peter M. Kloetzel

**Affiliations:** Charité – Universitätsmedizin Berlin, Corporate Member of Freie Universität Berlin and Humboldt-Universität zu Berlin, Institute of Biochemistry, Berlin, Germany

**Keywords:** proteasome, antigen processing, peptide splicing, adoptive T cell transfer, prediction algorithms, neosplicetopes

## Abstract

Proteasome generates spliced peptides by ligating two distant cleavage products in a reverse proteolysis reaction. The observation that CD8+ T cells recognizing a spliced peptide induced T cell rejection in a melanoma patient following adoptive T cell transfer (ATT), raised some hopes with regard to the general therapeutic and immune relevance of spliced peptides. Concomitantly, the identification of spliced peptides was also the start of a controversy with respect to their frequency, abundancy and their therapeutic applicability. Here I review some of the recent evidence favoring or disfavoring an immune relevance of splicetopes and discuss from a theoretical point of view the potential usefulness of tumor specific splicetopes and why against all odds it still may seem worth trying to identify such tumor and patient-specific neosplicetopes for application in ATT.

## Introduction

The majority of defined antitumor T cell responses involves the proteasomal processing of intracellular proteins and their presentation in the context of MHC class I molecules to peptide specific CD8+ T cells. Such antigenic peptides generated by the proteasome are 8-10 amino acids in length and mirror the linear sequence of the parental protein. Work over the past three decades has proven that the vast majority of these peptides are generated by the 26S proteasome, i.e. its 20S catalytic core as part of the ubiquitin proteasome system (UPS) (1–3).

One reason why the proteasome seems to be so ideally suited for the production of antigenic peptides is that its three active site β-subunits (β1s, β2s, β5s) (s-subunits) of the standard proteasome exhibit different cleavage specificities. These can be further modulated by replacing these s-subunits by alternative β1i, β2i and β5i immunosubunits (i-subunits) forming either immunoproteasomes (i-proteasomes) or by pairing with the standard β-subunits to form intermediate type 20S proteasomes. This allows proteasomes to cleave C-terminally of almost any of the 20 amino acids thereby meeting the diverse demands of the more than 10000 HLA class I allele variants for peptide binding (4–7). The combination of different active site β-subunits not only affects the cleavage site usage but also the cleavage strength of the 20S proteasome within a natural protein substrate (8, 9). In consequence, this provides the immune system with antigenic peptides of different linear sequences with different C-terminal anchor residues and also altered relative peptide abundancies that together affect the cellular immune response.

## Spliced Peptides, Epitopes of New Quality

**Figure 1:**
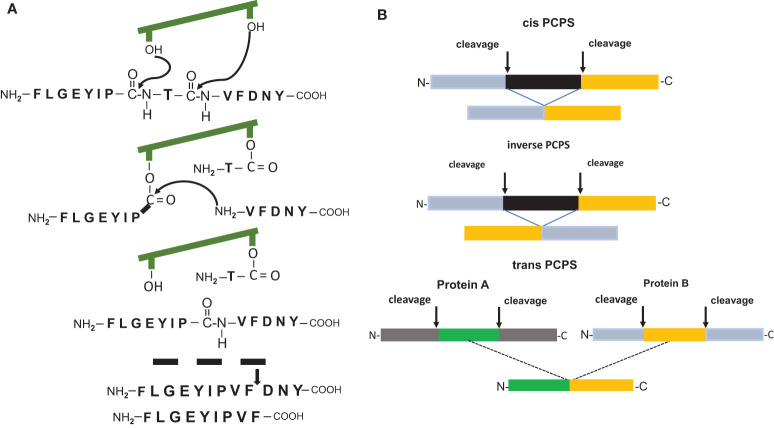
**(A)** Model of the peptide splicing reaction for the putative RAC2 P29L neosplicetope, adapted from Vigneron et al. (11). The spliced peptide requires an additional proteasomal cleavage for 9mer generation. **(B)** Proteasome catalyzed peptide splicing in cis, inverse and in trans.

It was undisputed that antigenic peptides of 8-12 amino acid residues in length generated by the 20S proteasome during the degradation of viral, bacterial or human proteins are peptide fragments with a linear sequence identical to that found in the unprocessed parental protein. However, two pioneering reports (10, 11) demonstrated the existence of HLA-1 bound CD8+ T cells reactive peptides which possessed an amino acid sequence that differed from that of the substrate protein and that were the result of a peptide splicing reaction. The spliced epitopes (splicetopes) were identified with the help of tumor patient derived T-cells and shown to be produced by the proteasome *via* a transpeptidation reaction. This involves the formation of an O-acyl-enzyme intermediate between a N-terminal peptide fragment and the Thr1 residue of one of the β-subunit active sites (**Figure 1A**). Thus, proteasome catalyzed peptide splicing (PCPS) represented a genuine novel catalytic function of the proteasome (11–15). Peptides can be spliced by PCPS in a cis or inverse order and theoretically even in trans, meaning that peptides derived from two different proteins are ligated and that the substrate proteins have to be present in the catalytic cavity of the 20S proteasome for degradation at the same time (**Figure 1B**). Importantly, the potential of splicetopes in cancer therapy was suggested by the fact that adoptive transfer of splicetope-specific CD8+ T cells into the autologous melanoma patient was shown to be followed by tumor regression (16). Moreover, CD8+ T cells directed against a spliced peptide expressed by human acute myeloid leukemia cells were shown to inhibit the engraftment of these leukemia cells in nonobese diabetic/severe combined immune deficient (SCID) mice (12). This data highlighted a potential immune relevance of such tumor antigen-derived splicetopes leading to the idea that establishing prediction algorithm aided pipelines for the CD8+ T cell independent identification of new splicetope may be a means to identify new targets for tumor therapy (17).

## Controversial Numbers

One issue which accompanied the identification T cell reactive splicetopes from the very beginning and which led to controversial discussions was the question on how frequent such splicetope producing splicing events indeed are, how abundant splicetopes are and whether spliced epitopes rather present a rare curiosity than being of genuine importance for the immune system. Initial calculations based on *in vitro* experiments estimated that epitope splicing efficacy ranged only between 0.0002% and 0.01% of the total proteasome-dependent epitope generation (11, 14, 18, 19) and that epitope production by PCPS was an extremely rare event. Indeed, one has to state that the number of verified proteasome-generated splicetopes derived from human tumor associated antigens that are recognized by CD8+ T cell still doesn´t exceed six and thus is the same number as almost twenty years ago. Aiming at a largescale identification of HLA-1 bound spliced epitopes (20) a complex algorithm-aided approach was developed to analyze HLA-1 immunopeptidome mass spectrometry data derived from the analysis of GR-LCL, CIR cell lines and human primary fibroblast. This analysis came to the exciting conclusion that approximately 25-30% of the HLA-1 immunopeptidome is comprised of spliced peptides, suggesting that spliced epitopes are by far more abundant than was previously estimated. Similarly, the application of a novel integrated bioinformatics workflow to analyze MS data and to discriminate between linear and spliced peptides eluted from HLA precipitates revealed a substantial contribution of both cis and trans spliced peptides to the immunopeptidome (21). Quite in contrast, by *de novo* sequencing and reanalyzing previously published data (22, 23) it was concluded that the amount of cis spliced peptides in the HLA-1 immunopeptidome ranges at most between 2-6% or only slightly above and are by far less prevalent than proposed. But even this considerably lower number was refuted in two recent reports. From the data it was concluded that peptides generated by PCPS do not play a major role among the HLA-1 presented peptides and amount to rather less than 1% of the HLA-1 immunopeptidome (24) or may not be generated at all (25). These analyses of the HLA-1 immunopeptidome severely questioned the general immune relevance of splicetopes and certainly seed doubts on the applicability of neosplicetopes for targeted TCR generation and adoptive T cell therapy. However, since some spliced peptides appear to be involved in tumor regression after adoptive T cell transfer (12, 16) this does not completely preclude their role in an anti-tumor response.

## Splicetopes as Potential Tool to Overcome Restrictions of Adoptive T-Cell Therapy

Adoptive T-cell therapy (ATT) is today the most effective form of immunotherapy. It involves the use of tumor-infiltrating lymphocytes (TILs), gene-modified T cells expressing a specific T cell receptor (TCR) and chimeric antigen receptor gene-modified T cells (CARs) (17, 26–29). Both, proteins with restricted expression (e.g. cancer/testis antigens (30) that are poorly or not expressed at all in normal adult tissues) and mutant proteins can be recognized by TCRs. When specific TCRs are used to target cancer cells, the quality of the target epitope (quantity and affinity to HLA-1 molecules) and the quality of the T cells (avidity) determine whether or not a tumor is eradicated by T cells expressing tumor-reactive TCRs (31). Until recently, mostly non-mutated shared tumor-associated (self-) antigens (TAAs) have been used as targets in ATT trials. However, having undergone thymic selection many epitopes derived from these self-antigens possess low affinities to the patients’ HLA-molecules and the immune response is not sufficient for long-term eradication of tumors. In addition, many of these antigens are not driving the oncogenic events and their expression is therefore not essential for cancer cell outgrowth. Ideally, many of these problems could be avoided by targeting so called tumor neoantigens that harbor somatic driver mutations whose expression is crucial for the survival of cancer cells and which are not expressed in non-tumor cells. For example, long term responses in melanoma patients correlated with neoantigen specific T cells in the *in vitro* expanded and infused tumor infiltrating lymphocytes (31). Neoantigens thus represent critical targets for effective anti-tumor T-cell responses and for eradication of advanced cancers with respect to both, efficacy and safety (32–36).

Although targeting cancer-specific neoepitopes by TCR-mediated adoptive T cell transfer (ATT) represents a very promising approach for personalized cancer therapy (26, 33, 36) there are several limitations which may restrict their broad application of cancer immunotherapy. For example, often neoepitopes will not exhibit HLA class I binding affinities sufficient to trigger an efficient T cell response or will not be generated efficiently by the proteasome. In fact, many if not most of the mutations within neoantigens will not be part of or lead to the generation of high-affinity neoepitopes (36). For example, frequent recurrent mutations involving the same amino acid, such as the KRAS G12V mutation do not fit into the most frequent HLA-alleles of the general population, i.e. the HLA-A*02:01 allele which can be found in about 40% of Caucasians. Even if a suitable neoepitope is generated, its haplotype specificity frequently does not match with the patient´s HLA class I allele, consequently excluding these tumor patients from ATT. Another example is the adaptor protein MyD88. A missense mutation almost consistently changing leucine in position 265 to proline (L265P) is one of the most common driver mutations found in about one-fifth of all lymphoid malignancies (37–40). The resulting MyD88 L265P derived neo-epitope however exclusively binds to HLA-B*07:02. Therefore, only patients that carry the HLA-B*07:02 haplotype will potentially benefit from ATT by using a recently published MyD88 L265P specific TCR (41). So far, problems like these prevented the large-scale use of mutation-specific TCRs using T cells directed against mutational epitopes. Theoretically, given that peptide splicing leads to the generation of peptides containing distant fragments of proteins, PCPS harbors the potential to generate mutant spliced peptides with slightly altered sequences that bind efficiently to patient HLA-1 and might therefore represent more suitable TCR targets. This in particular in those cases where the recurrent somatic mutations in a tumor antigen do not lead to a non-spliced unique tumor neoepitope either exhibiting a sufficient HLA class I binding affinity or the appropriate HLA class I haplotype (36). Thus, spliced neoepitopes, i.e. neosplicetopes containing the tumor specific mutation, harbor the theoretical potential to extend the haplotype diversity or HLA-1 binding affinity of tumor specific epitopes for generation of tumor and patient specific TCRs. In theory, PCPS may thus represent an interesting novel approach. However, the major problem connected with it is to develop a method allowing the reliable identification of such spliced mutation harboring neosplicetopes.

## Trying a Reverse Immunology Pipeline

In light of the above results and conclusions drawn from the immunopeptidome analyses, the search for splicetopes within the HLA-1 immunopeptidome for application in tumor therapy may seem like looking for a needle in the haystack. Nevertheless, the identification of tumor patient derived CD8+ T-cells recognizing splicetopes and their successful application in tumor therapy by ATT indicates that peptide splicing may not be such an accidental event and that proteasomal generation of tumor cells derived splicetopes represents an ordered, non-random event of sufficient repeatability and frequency. Furthermore, following the outlined theoretical arguments that identification of suitable tumor specific neosplicetopes for ATT may allow to make neoantigens visible for the immune systems which cannot be recognized otherwise alternative approaches allowing a more direct splicetope seemed worthy to consider. With this in mind the spliced peptide predictions algorithms ProteaJ and ProtAG (42, 43) were developed. In combination with mass spectrometric analyses of *in vitro* PCPS assays these permit the identification spliced peptides that are generated by the 20S proteasome from synthetic polypeptide substrates. It is well established that the *in vitro* generation of “conventional” non-spliced linear epitopes from polypeptides substrates harboring viral, bacterial or tumor antigen derived epitopes often reflect the *in vivo* situation of antigen processing with high fidelity both with regard to peptide sequence and amounts (44–51). Alike the fidelity of the generation of linear epitopes, *in vitro* PCPS experiments showed that published immune reactive spliced epitopes, derived from fibroblast growth factor FGF-5, the melanocyte growth protein gp100^mel^, tyrosinase and the SP110 nuclear protein were also efficiently generated by *in vitro* PCPS assays (12, 18, 19, 51–53). In fact, the *in vitro* PCPS assays suggested that splicetopes that are recognized by CD8+ T cells on the cell surface were produced with amounts reminiscent of what is obtained when virus or tumor derived non-spliced linear epitopes are generated by purified 20S proteasomes *in vitro* from synthetic peptide substrates. Furthermore, *in vitro* application of the prediction algorithm ProteaJ allowed the *de novo* identification of a gp100^mel^ derived spliced epitope eliciting a peptide specific T cell response (53). Interestingly, judging by the number of different spliced peptides generated in an *in vitro* PCPS reaction the proteasomal splicing reaction as such appears to be a relatively frequent event during proteasomal peptide hydrolysis (15). The relative number of different spliced peptides generated in an *in vitro* PCPS reaction can however vary considerably between different *in vitro* substrates indicating that the monitored proteasomal peptide splicing frequency is sequence dependent (15). This conclusion is underlined by a recent study in which a large number of different substrates were studied *in vitro* in order to determine sequence motives that support proteasomal splicing reactions (54) as well as by the observation that proteasome subtypes with slightly different cleavage site preferences also differ in their peptide splicing efficiencies (51, 54). Underlining the feasibility of an algorithm aided “reverse immunology” approach, two high affinity splicetopes within the secreted bacterial phospholipase PlcB priming antigen-specific CD8^+^ T cells in *L. monocytogenes*-infected mice were successfully identified (55). Corroborating experimental data with tumor derived splicetopes both PlcB derived splicetopes were also generated by PCPS *in vitro*. These experiments were the first to show that PCPS expands the CD8^+^ T cell response against L. monocytogenes by exposing splicetopes on the cell surface.

Even though the analyses of immunopeptidomes failed to identify relevant numbers of spliced epitopes, the observed fidelity of the *in vitro* splicing reaction in generating known or predicted immune responsive splicetopes suggested that the application of *in vitro* PCPS assays in combination with prediction algorithms facilitating the identification of splicetopes or neosplicetopes by mass spectrometry may be worthy to test in search for new immune relevant neosplicetopes.

## A Pipeline of Low Fidelity

In a proof of principle study Willimsky et al. (43), tested whether an experimental approach solely based on epitope prediction by algorithms or in combination with *in vitro* PCPS can successfully be applied for the identification of novel neosplicetopes from mutKRASG12V and the mutant RAC2-P29L neoantigens for consecutive generation of splicetope specific TCRs. Both are a so-called driver mutation facilitating tumor growth as well as metastasis and thus present potential targets in ATT. The oncogenic KRASG12V mutant had been chosen because the recurrent G12V mutation does not result in the formation of a high affinity HLA-A*02:01 non-spliced neoepitope. mutRAC2 was selected because the P29L mutation is part of a high affinity HLA-A*02:01 neoepitope and the generation of a putative mutRAC2 derived neosplicetope could therefore be studied in comparison to proteasomal generation of the linear neoepitope. *In silico* analysis of the mutRAC2 neoantigen predicted a single RAC2-P29L derived putative neosplicetope with high affinity for binding. However, although this putative RAC2-P-29L derived neosplicetope was generated in *in vitro* PCPS assays, a high affinity TCR generated against this putative mutRAC2 neosplicetope in an established humanized mouse model (56) failed to indicate any immune relevance or to detect any cell surface presentation of this spliced peptide. For comparison the non-spliced mutRAC2 neoepitope was abundantly generated *in vitro* and efficiently recognized by the corresponding TCR at the cell surface. These combined biochemical/immunological explorative experiments suggest that the predicted mutRAC2 neosplicetope is either not generated *in vivo* or its amounts are too low to trigger a T-cell response even when the mutRAC2 neoantigen is overexpressed. *In silico* analysis of the mutKRASG12V neoantigen predicted three putative neosplicetopes with high HLA-A*02:01 binding affinities. However, TCRs generated against these peptides based on *in silico* predictions failed to demonstrate their immune relevance in T cells assays. Other than reported for a previously proposed *in silico*-*in vitro* pipeline for the identification of neosplicetopes (57) also the *in vitro* generation of the proposed mutKRASG12V derived neosplicetopes could not be validated (43). In addition, applying targeted mass spectrometry and using heavy isotope labeled peptides the identification of the proposed neosplicetopes was proven to be false (58, 59).

Why then did the identification of a mutRAC2 neoantigen derived neosplicetope fail despite its generation by *in vitro* PCPS? One obvious explanation is that *in silico* prediction and/or *in vitro* PCPS are not reliable predictors for the *in vivo* generation of a splicetope. On the other hand, taken all available experimental evidence (see above) there appears little reason to assume that the 20S proteasome splicing activity as such differs between the *in vitro* and *in vivo* situation. What most likely might differ is the efficiency at which a spliced peptide is generated either *in vitro* or *in vivo.* One also has to consider that in order to facilitate their mass spectrometric detection *in vitro* splicing reactions are often driven by increased substrate concentrations (15) potentially leading to a false impression of the actual splicetope generation efficiency. Due to the high substrate density within the catalytic cavity when synthetic polypeptide substrates are used one also cannot entirely exclude that under *in vitro* conditions splicing events are enforced which *in vivo* will not occur at all or less frequently than *in vivo*, when the amino acid sequence motif supporting the generation of the spliced epitope is present within the proteasomal catalytic cavity only once. Furthermore, the proteasomal transpeptidation reaction is most likely less frequent than the normal peptide hydrolysis reaction that results in the generation of non-spliced peptide fragments including linear HLA-1 epitopes. This latter point is nicely reflected by the analysis of mutRAC2P29L where *in vitro* generation of the linear spliced mutRCA2 derived neoepitope is at least 200fold more efficient than the generation of the putative mutRAC2 neosplicetope (43). From these studies we have to acknowledge that an *in silico-in vitro* pipeline thought to be a straight forward, easy to use approach to identify spliced peptides for therapeutic use cannot be the method of choice (57) and that *in silico* predictions of spliced epitopes alone or in combination with algorithms calculating HLA-1 binding affinity (60) and *in vitro* PCPS reactions seem by no means of sufficient reliability to set out for laborious and time consuming TCR generation.

## Conclusions

Taken the available experimental data and in light of the mass spectrometric immunopeptidome analyses (22–25) one could argue that due to the limited cell surface expression of spliced epitopes a search for immune relevant neosplicetope is in vain. On the other hand, considering the therapeutic potential of adoptive T cell transfer for patient specific tumor treatment (35, 36), and the above outlined potential difficulties in finding patient specific appropriate neoepitopes for TCR generation, neosplicetopes generated by PCPS might still carry the potential to overcome some of the restrictions connected with ATT.

Reconsidering the identification of splicetope recognizing T cells derived from melanoma patients (16) one possible way to validate a potential neosplicetope is to isolate tumor patient derived neosplicetope reactive T cell clones in tumor infiltrating lymphocytes for neosplicetope reactivity. However, the number of patients possessing the correct HLA haplotype in combination with a suitable somatic mutation in a tumor specific neoantigen will most likely limit a wide scale application. Alternatively, one may try to generate neosplicetope reactive T cell clones *in vitro* or in experimental humanized mouse models (56) in order to isolate neosplicetope-specific TCR. However, isolating a T cell clone against a peptide is itself not sufficient to unequivocally demonstrate that this peptide indeed exists and is processed by the tumor. What finally will be needed is experimental evidence that the isolated T cell clone or transduced T cells that express the isolated TCR are able to recognize or to kill tumors expressing the HLA molecules presenting the peptide and the neoantigen from which the putative neosplicetope is derived. One also has to consider that proteasomal generation of neosplicetopes is the result of peptide shuffling bearing the possibility that the generated neosplicetopes are not entirely tumor specific. By setting the search window of the spliced peptide prediction algorithms in way, that neosplicetopes are predicted in which only one or two amino acids are exchanged by the splicing reaction one can significantly minimize the likelihood of cross reactivity. At the end it will depend entirely on the specificity of the generated TCR, which always requires extensive testing because cross reactivity never can be excluded to 100%.

Thus, before starting the screen and despite all caveats one may therefore still want to demonstrate that the *in silico* predicted neosplicetope is also generated *in vitro.*

To circumvent the pitfalls connected with the strictly *in silico* and *in vitro* based prediction of neosplicetope for TCR generation a á priori experimental proof that *in silico* predicted neo-splicetopes are generated *in vivo* and expressed at the cell surface in the context of HLA-1 proteins may seem to be mandatory. However, identification of predicted neosplicetopes among the large population non-spliced peptides eluted from immunoprecipitated HLA-1 molecules will be challenging. It will need the development of a new mass spectrometry compatible algorithm in combination with highly sensitive targeted mass spectrometry as recently described by Beer for KRAS G12V derived peptides (58). For identification by targeted LC-MS/MS predicted spliced peptides have to be synthesized with heavy isotope labeled amino acids and spiked into the eluted peptide preparation before mass spectrometric analysis in order to permit unequivocal validation of the putative neosplicetope. Neither experimental approach represents a straight forward pipeline for spliced epitope identification and may turn out to document that spliced epitopes or neosplicetopes are only of theoretical immune relevance and of theoretical therapeutic potential. However, despite all odds if successful and in light of the expected gain it may still seem worth a try.

## Author Contributions

The author confirms being the sole contributor of this work and has approved it for publication.

## Funding

Part of this work was supported by grants from the Berlin Institute of Health (CRG-1), Einstein Stiftung (A- 2013-174) and Berliner Krebsgesellschaft.

## Conflict of Interest

The author declares that the research was conducted in the absence of any commercial or financial relationships that could be construed as a potential conflict of interest.

## Publisher’s Note

All claims expressed in this article are solely those of the authors and do not necessarily represent those of their affiliated organizations, or those of the publisher, the editors and the reviewers. Any product that may be evaluated in this article, or claim that may be made by its manufacturer, is not guaranteed or endorsed by the publisher.
